# Metabolism and pharmacokinetics of a novel polyphenol fatty acid ester phloridzin docosahexaenoate in Balb/c female mice

**DOI:** 10.1038/s41598-020-78369-0

**Published:** 2020-12-07

**Authors:** Wasundara Fernando, Kerry B. Goralski, David W. Hoskin, H. P. Vasantha Rupasinghe

**Affiliations:** 1 Department of Pathology, Faculty of Medicine, Dalhousie University, Halifax, NS Canada; 2 Department of Pharmacology, Faculty of Medicine, Dalhousie University, Halifax, NS Canada; 3 Department of Pediatrics, Faculty of Medicine, Dalhousie University, Halifax, NS Canada; 4 Department of Microbiology and Immunology, Faculty of Medicine, Dalhousie University, Halifax, NS Canada; 5 Department of Surgery, Faculty of Medicine, Dalhousie University, Halifax, NS Canada; 6 College of Pharmacy, Dalhousie University, Halifax, NS Canada; 7 Department of Plant, Food, and Environmental Sciences, Faculty of Agriculture, Dalhousie University, Truro, NS Canada; 8 Division of Hematology/Oncology, IWK Health Centre, Halifax, NS Canada

**Keywords:** Cancer, Drug discovery

## Abstract

Flavonoids are known to undergo phase II metabolism and produce metabolites with similar or stronger biological effects compared to the parent flavonoids. However, the limited cellular uptake and bioavailability restrict their clinical use. We synthesized phloridzin docosahexaenoate (PZ-DHA), a novel fatty acid ester of polyphenol, through an acylation reaction with the aim of increasing the cellular availability and stability of the parent biomolecules, phloridzin (PZ) and docosahexaenoic acid (DHA). Here, we report metabolites and pharmacokinetic parameters of PZ-DHA, determined using ultra-high-performance liquid chromatography-electrospray ionization tandem mass spectrometry. PZ-DHA was taken-up by human (MDA-MB-231, MDA-MB-468, and MCF-7) and mouse (4T1) mammary carcinoma and human non-malignant mammary epithelial cells (MCF-10A) in cellular uptake assays. Our results suggested that the acylation improves the cellular uptake of PZ and stability of DHA within cells. In mouse hepatic microsomal assays, two major glucuronides of PZ-DHA, PZ-DHA-4-*O*-glucuronide and PZ-DHA-4′-*O*-glucuronide (MW = 923.02 g/mol), were detected. One tri-methylated- (4,4′,6′-O-trimethyl-PZ-DHA) (MW = 788.88 g/mol) and one di-sulphated- (PZ-DHA-4,4′-O-disulphide) PZ-DHA metabolite (MW = 906.20 g/mol) were also identified. Intraperitoneal injections of PZ-DHA (100 mg/kg) into Balb/c female mice was rapidly absorbed with a serum C_max_ and T_max_ of 23.7 µM and 60 min, respectively, and rapidly eliminated (t_1/2_ = 28.7 min). PZ-DHA and its metabolites are readily distributed throughout the body (V_d_ = 57 mL) into many organs. We identified in vitro and in vivo metabolites of PZ-DHA, which could be tested for potential use to treat diseases such as cancer in multiple organ systems.

## Introduction

The establishment of pharmacokinetic parameters is a necessary first step in the early stages of drug development^1^. Pharmacokinetics explains the absorption, distribution, metabolism, and excretion of drugs/xenobiotics introduced into the body. The pharmacological activity of a drug depends on its ability to reach the target site, as well as being available in adequate concentrations to mediate an effect. Even though the dose largely determines the availability of a drug in the systemic circulation and at the target site of action, the dose is not the sole determinant of these parameters^2,3^. Absorption, tissue distribution, phase I and II metabolism, and biliary and renal excretion are also critical contributing factors toward the pharmacological activity of a drug. In addition, drug-induced toxicity and adverse side effects may be explained by impaired distribution, metabolism and/or excretion of a particular drug^1,4–6^. Phase I biotransformation (often referred to as phase I metabolism) includes de novo formation or exposure of functional groups on drugs/xenobiotics, resulting in increased hydrophilicity and/or polarity. These reactions are governed by cytochrome-p450 (CYP450) enzymes acting as monooxygenases, dioxygenases, and hydrolases^7,8^. Phase II metabolism processes drugs/xenobiotics into easily excretable forms; therefore, phase II reactions (also known as conjugation reactions) play a leading role in detoxifying transformations^9,10^.

Flavonoids are a sub-group of plant polyphenols that are known to undergo phase II metabolism. In some cases, the metabolites are active and show similar or stronger biological effects compared to the parent flavonoids^11–14^. However, the clinical applications of flavonoids are restricted by their poor cellular uptake and bioavailability; therefore, attempts have been made to synthesize flavonoid derivatives, aiming to overcome these limitations^15^. With this objective in mind, phloridzin docosahexaenoate (PZ-DHA) was synthesized by acylation of phloridzin (PZ), a flavonoid precursor, with docosahexanoic acid (DHA), an omega-3-fatty acid, through an enzyme-catalyzed esterification reaction^16^. In our previous studies, we have shown selective cytotoxic activity of PZ-DHA against hepatocellular carcinoma^17^, breast cancer^18^, leukemia^19^, and skin cancer^20^ cells. Recently, we demonstrated that intraperitoneal administration of PZ-DHA inhibits the metastasis of breast cancer cells into the lungs of Balb/c and non-obese diabetic severe combine immunodeficient female mice^21^. In the current study, the ability of PZ-DHA to penetrate the cell membrane of mammary carcinoma cells and non-malignant mammary epithelial cells, as well as the stability of PZ-DHA in the cellular environment over a prolonged period, was studied. For comparison purposes, the parent compounds of PZ-DHA were also included in the experiments. Phase I and phase II metabolites of PZ-DHA were identified by incubating PZ-DHA with freshly-prepared mouse hepatic microsomal preparations in the presence or absence of phase II co-factors. In addition, the biological fate of PZ-DHA was investigated following the intraperitoneal administration of PZ-DHA to Balb/c female mice.

## Results

### Acylation of DHA to PZ increases the cellular uptake of PZ and stabilization of DHA

**Figure 1:**
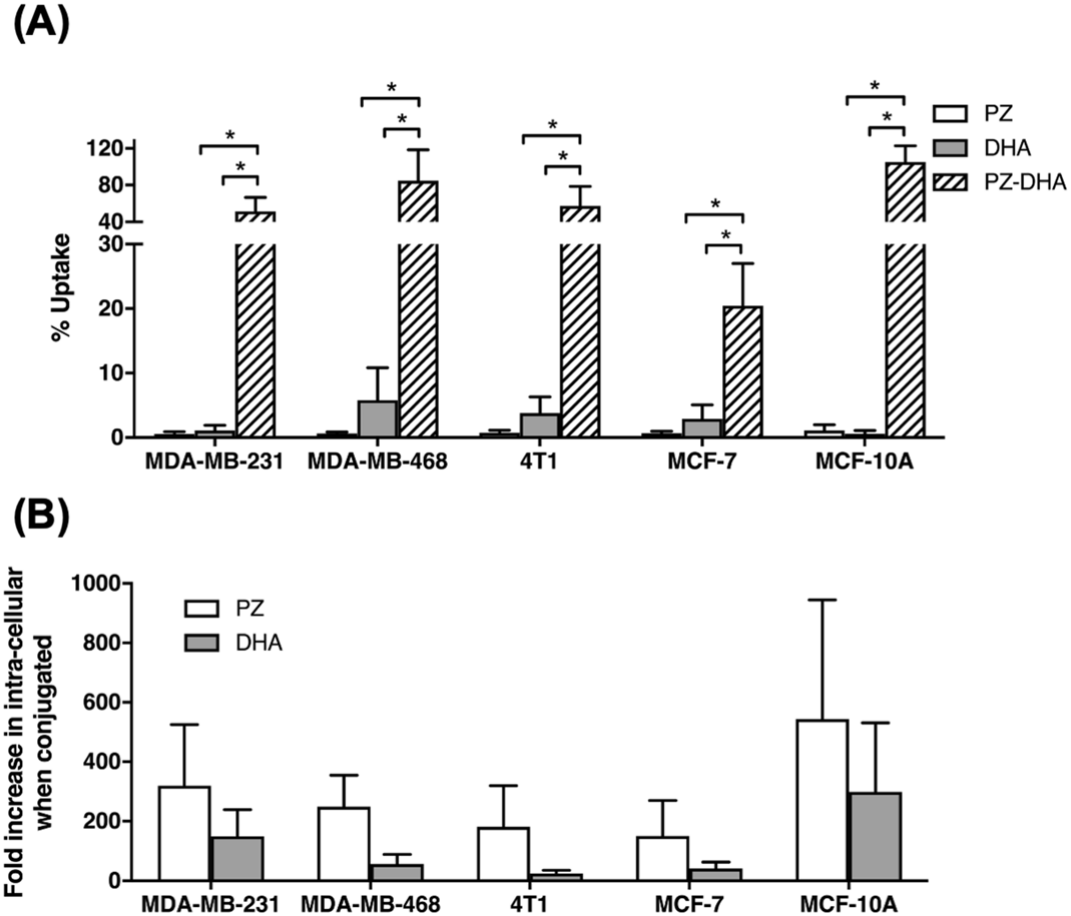
Conjugation of PZ with DHA increases the cellular uptake and stability of PZ and DHA. The cells were treated with 20 μM of PZ, DHA or PZ-DHA and cultured for 72 h at 37 °C. Cells were harvested and washed thoroughly using cold PBS. Cells were lysed in cold acetone containing 0.05 mg/mL quercetin, and cell lysates were collected by centrifugation and analyzed by using UPLC-ESI-MS/MS. **(A)** mean % cellular uptake of PZ, DHA and PZ-DHA. (**B**) fold increase in intracellular PZ and DHA when conjugated. ANOVA multiple means statistical comparison method was performed and differences among means were compared using Tukey’s test; *p < 0.05. (MDA-MB-231 *p* = 0.008; MDA-MB-468 *p* = 0.01; 4T1 *p* = 0.02; MCF-7 *p* = 0.01; MCF-10A *p* = 0.0005).

Determination of PZ, DHA, and PZ-DHA uptake by mammary carcinoma cells and mammary epithelial cells was performed using ultra-high-performance liquid chromatography-electrospray ionization- tandem mass spectrometry (UPLC-ESI–MS/MS) analysis of cell lysates made in acetone followed by reconstitution in methanol. A 0.25 mg/mL standard of PZ, DHA or PZ-DHA made in methanol was used to determine the retention times (RT) for PZ (1.03 min), DHA (10.37 min) and PZ-DHA (9.04 min) using a Resteck biphenyl column. In all cell lines, the uptake (as a % of the dose) was very low for PZ and DHA. The cellular uptake of PZ-DHA was significantly higher than that of PZ or DHA in all cell lines (Fig. 1A). In the malignant cells, PZ-DHA uptake ranged from 21% (MCF-7 cells) to 50% (MDA-MB-468 cells) but was not significantly different across malignant cell types. When administered as PZ-DHA, the intracellular fold-increase in PZ ranged from 150-fold (MCF-7 cells) to 320-fold (MDA-MB-231 cells) and for DHA 25-fold (4T1 cells) to 150-fold (MDA-MB-231 cells); fold-increase values were not statistically different across malignant cell types (Fig. 1B). PZ-DHA uptake and fold-increase in intracellular PZ and DHA were similar in the non-cancerous MCF-10A cells versus the malignant cells.

### PZ-DHA undergoes phase I and II metabolism in vitro

**Figure 2:**
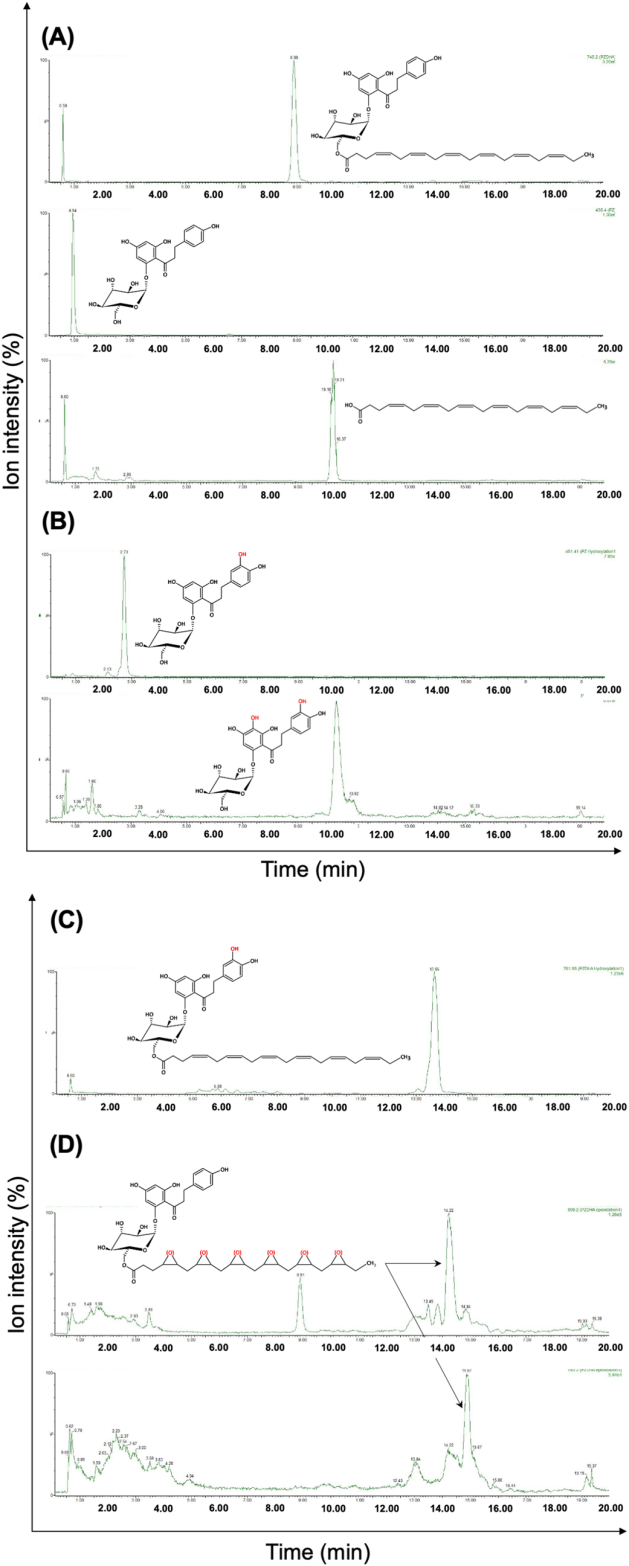
PZ-DHA undergoes phase I metabolism in the presence of freshly prepared mouse liver microsomes. PZ-DHA (1.1 µM) was incubated with 100 mM NADPH in the presence of mouse liver microsome for 1 h at 37 °C. Metabolites from PZ-DHA **(A)** hydrolysis, **(B)** and **(C)** hydroxylation, and **(D)** epoxidation were identified.

Five potential phase I reactions, namely, hydrolysis of PZ-DHA, hydroxylation of PZ, hydroxylation of PZ-DHA, epoxidation of DHA, and epoxidation of PZ-DHA were predicted based on the chemical structure of PZ-DHA and most likely potential phase I metabolic conversions of flavonoids and fatty acids. The molecular weights of these predicted metabolites were calculated and tested. In hepatic microsomal assays, metabolites matching the molecular masses of PZ (MW = 436.4 g/mol) and DHA (MW = 328.47 g/mol) were identified as the products of hydrolysis of PZ-DHA (Fig. 2A). Two hydroxylated metabolites of PZ (3-hydroxy-phloridzin, MW = 451.41 g/mol; 3,5′-dihydroxy-phloridzin, MW = 467.41 g/mol) (Fig. 2B) and one mono-hydroxylated metabolite of PZ-DHA (3-hydroxy-phloridzin docosahexaenoate, MW = 761.88 g/mol) (Fig. 2C) were identified. Two epoxy metabolites of PZ-DHA were also identified; however, collected data were not adequate to predict the precise sites of epoxidation on the long carbon chain (tri-epoxy-phloridzin docosahexaenoate, MW = 793.2 g/mol; tetra-epoxy-phloridzin docosahexaenoate, MW = 809.2 g/mol) (Fig. 2D). No epoxy-DHA metabolites were detected during the study.

**Figure 3:**
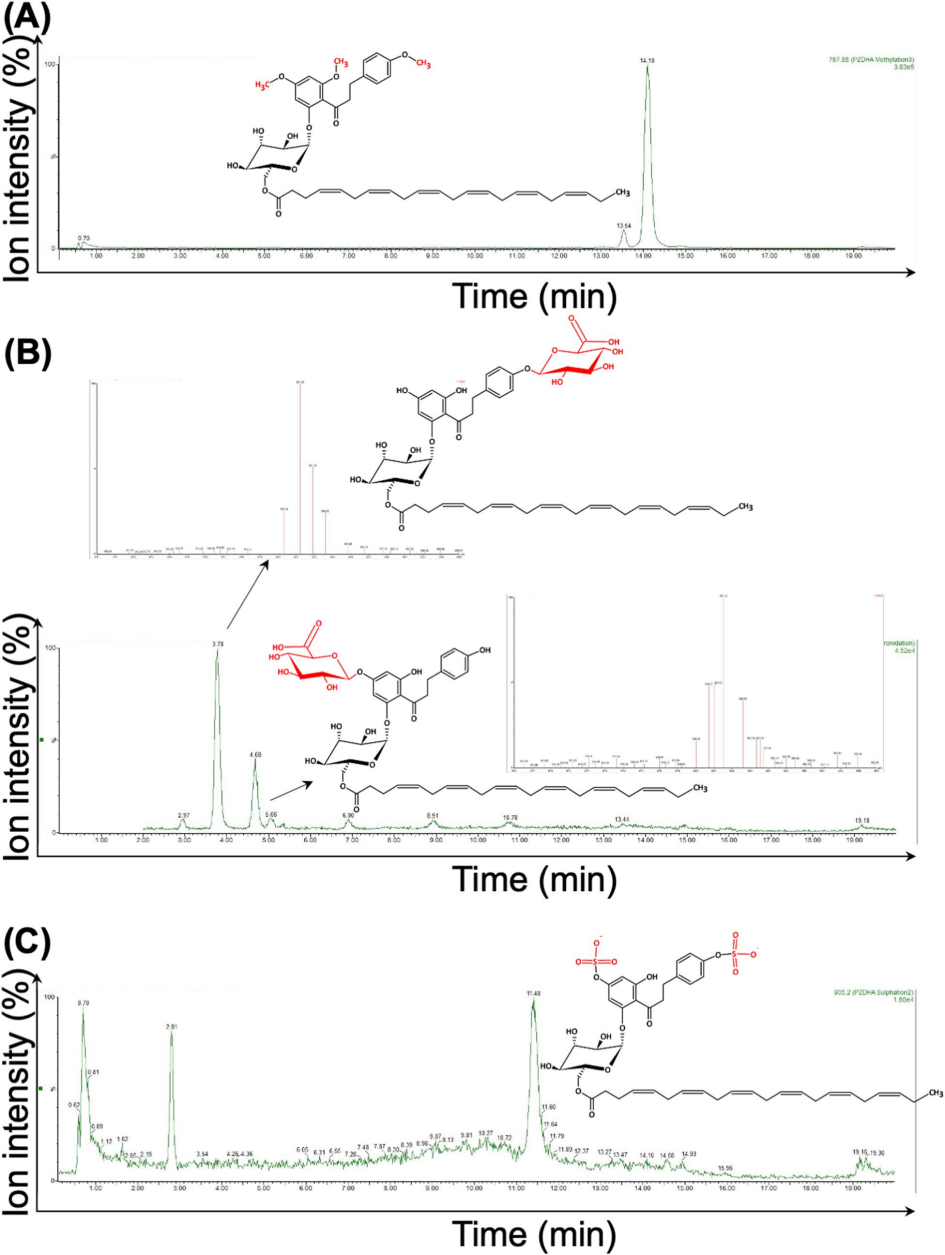
PZ-DHA undergoes phase II metabolism in the presence of freshly prepared mouse liver microsomes. PZ-DHA (1.1 µM) was incubated with *S*-(5′-adenosyl)-*L*-methionine chloride dihydrochloride (1 mM), glucuronic acid (2 mM) and alamethicin (25 µg/mL) and adenosine 3′-phosphate 5′-phosphosulfate lithium salt, hydrate (0.5 mM) in the presence of mouse liver microsomal enzymes (3 mg/mL) at 37 °C for 1 h. **(A)** Tri-methylated-PZ-DHA (4,4′,6′-tri-*O*-methyl-phloridzin docosahexaenoate) (MW = 788.88 g/mol), **(B)** phloridzin docosahexaenoate-4-*O*-glucuronide (MW = 923.02 g/mol) and phloridzin docosahexaenoate-4′-*O*-glucuronide (MW = 923.02 g/mol), and **(C)** sulfated-PZ-DHA (phloridzin docosahexaenoate-4,4′-di-*O*-sulphide) metabolite (MW = 906.20 g/mol) were identified.

Phase II metabolites of PZ-DHA were identified by incubating PZ-DHA with S-adenosylmethionine, UDP-glucuronic acid or 3′-phosphoadenosine-5′-phosphosulfate in the presence of freshly prepared mouse hepatic microsomal preparations. One methylated-metabolite (4,4′,6′-tri-*O*-methyl-phloridzin docosahexaenoate, MW = 788.88 g/mol, RT = 14.10 min) (Fig. 3A), two glucuronides (phloridzin docosahexaenoate-4-*O*-glucuronide, MW = 923.02 g/mol, RT = 3.78 min and phloridzin docosahexaenoate-4′-*O*-glucuronide, MW = 923.02 g/mol, RT = 4.68 min) (Fig. 3B) and one sulfated metabolite (phloridzin docosahexaenoate-4,4′-di-*O*-sulfide MW = 906.20 g/mol, RT = 11.40 min) (Fig. 3C) were identified.

### PZ-DHA is absorbed through intraperitoneal route and undergoes phase I and phase II metabolism in Balb/c female mice

**Figure 4:**
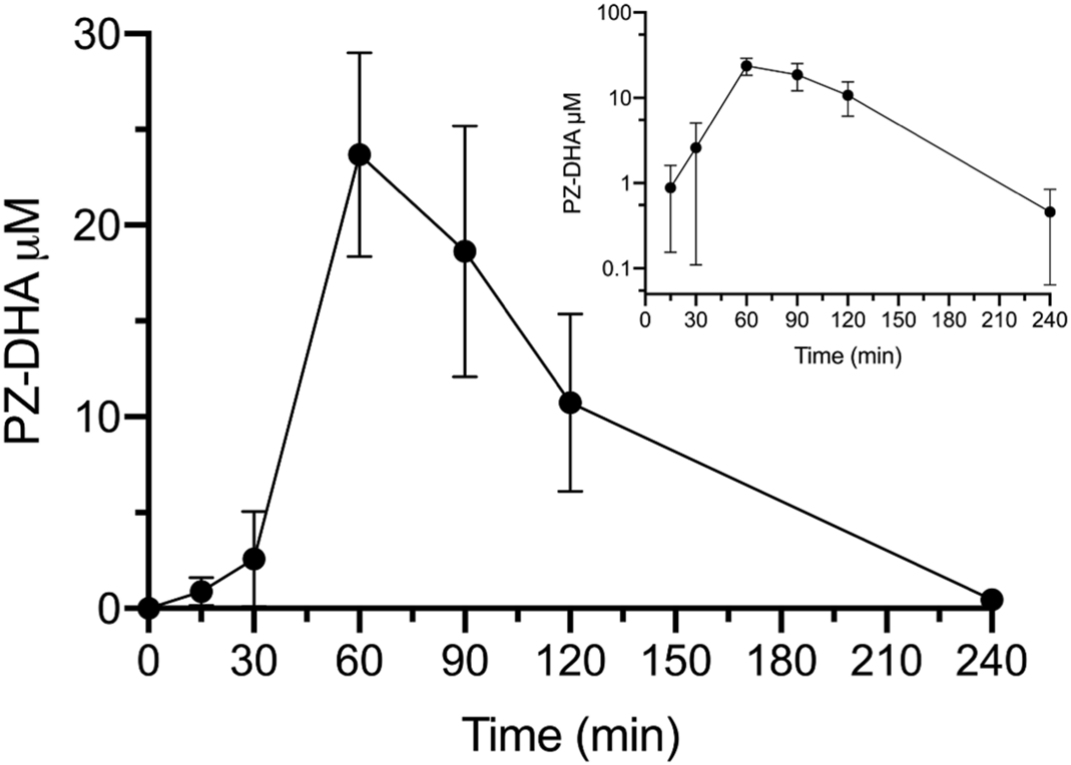
Intraperitoneal administration of PZ-DHA results in absorption into the systemic circulation of Balb/c female mice. PZ-DHA (100 mg/kg) was administered by intraperitoneal injection to Balb/c female mice. Blood collection was performed and serum was separated. PZ-DHA was identified and quantified using a calibration curve (R^2^ = 0.99) generated using a standard series of PZ-DHA concentrations made in mouse serum. PZ-DHA concentration was plotted against time. The elimination rate of PZ-DHA was determined using a semi-log curve of PZ-DHA concentration vs. time curve.

**Figure 5:**
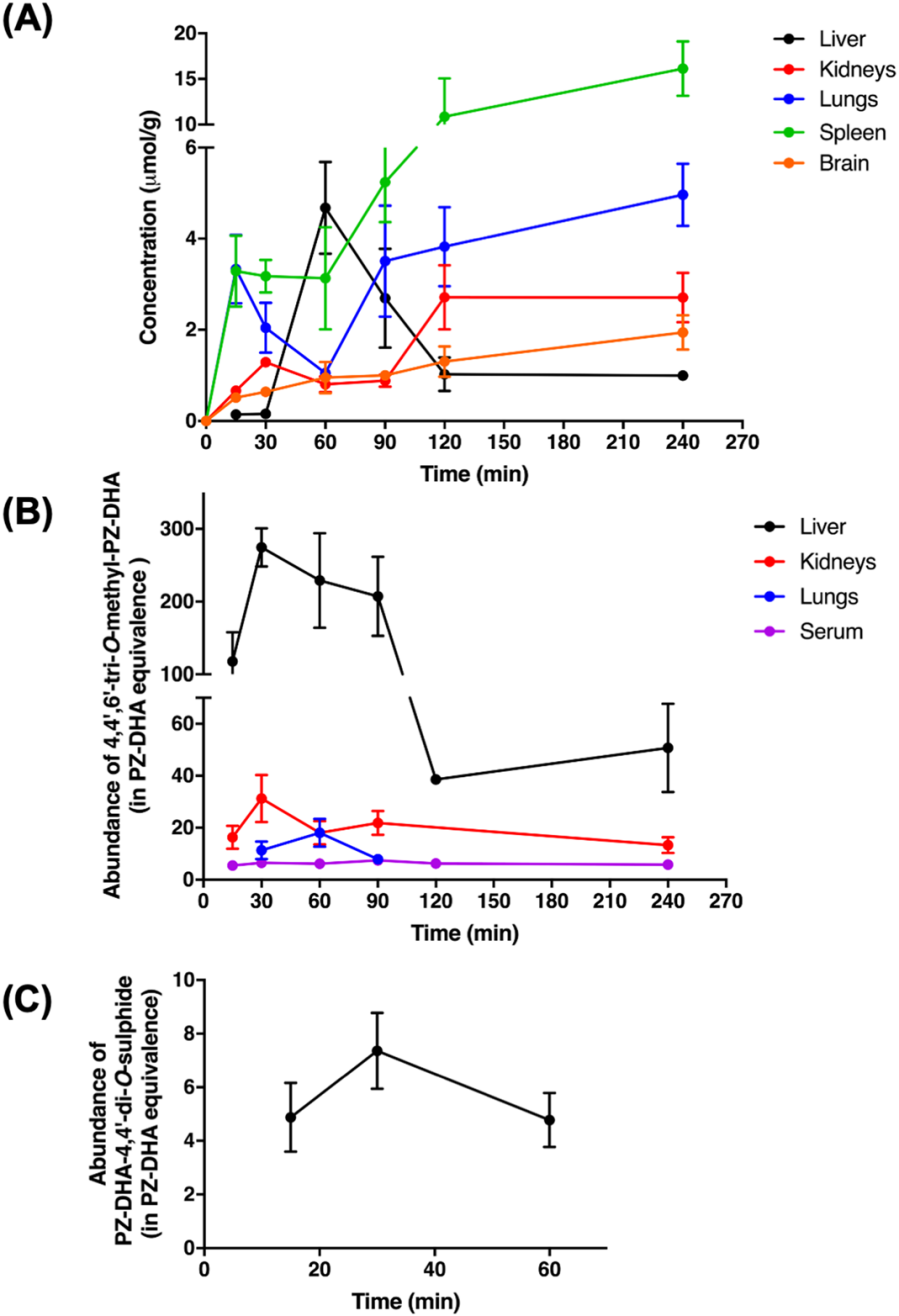
PZ-DHA and metabolites were detected in various organs of PZ-DHA-treated Balb/c female mice. PZ-DHA (100 mg/kg) was administered by intraperitoneal injection to Balb/c female mice and liver, lungs, kidneys, spleen and brain were harvested. Organs were homogenized and PZ-DHA in **(A)** liver, kidneys, lungs, spleen, and brain was identified and quantified using a calibration curve. The abundance of **(B)** 4,4′,6′-tri-*O*-methyl-phloridzin docosahexaenoate in liver, kidneys, lungs, and serum) and **(C)** phloridzin docosahexaenoate-4,4′-di-*O*-sulphide in the liver was determined in PZ-DHA equivalence.

Following intraperitoneal (IP) administration, PZ-DHA was rapidly absorbed into the systemic circulation (Fig. 4). PZ-DHA was detectable (0.9 ± 0.4 µM) at the 1^st^ measured time point (15 min) and reached its maximum serum concentration (C_max_) of 23.4 μM after 60 min (t_max_) (Table 1). Thereafter, the amount of serum PZ-DHA declined linearly with a t_1/2_ = 28.7 min but remained detectable to the last measured time (240 min). Phase I metabolism of PZ-DHA in mice was evident by the detection of PZ and DHA in serum. Other phase I metabolites were not detected in serum. PZ-DHA gradually accumulated in the lungs, kidneys, spleen, and brain with respective maximal detected concentrations per gram of tissue of 4.9 ± 0.7 µmol/g, 2.7 ± 0.5 µmol/g, 16.1 ± 2.9 µmol/g, and 1.9 ± 0.4 µmol/g after 240 min (Fig. 5A). As anticipated, PZ-DHA concentration in the liver increased during the absorption phase (15–60 min), reaching a maximal detected concentration of 4.7 ± 1.1 µmol/g, and decreased in the elimination phase (60–240 min) of PZ-DHA. Continuous increases in PZ-DHA tissue/serum ratio in the PZ-DHA serum elimination phase are consistent with gradual tissue accumulation of PZ-DHA over time (Table 2). Furthermore, a tri-methylated PZ-DHA phase II metabolite (4,4′,6′-tri-*O*-methyl-phloridzin docosahexaenoate) was also quantified in comparison to PZ-DHA concentrations in each tissue. This metabolite was detected in the liver, lungs, and kidneys but was not found in the spleen or brain; 4,4′,6′-tri-*O*-methyl-phloridzin docosahexaenoate reached the maximum concentration before 1 h post-injection in all organs except for serum in which the concentration remained constant (Fig. 5B) throughout the experimental period. The di-sulfide metabolite of PZ-DHA, phloridzin docosahexaenoate-4,4′-di-*O*-sulphide, was also studied; it was detected in the liver for a short period of time but was not found in any other organ or in serum at a detectable concentration (Fig. 5C).

**Table 1:** Pharmacokinetic parameters of PZ-DHA in Balb/c female mice.

Pharmacokinetic parameter	Symbol	Estimated value
Maximum serum concentration	C_max_	23.4 µM
Time to reach maximum serum concentration	T_max_	60 min
Biological half-life	T_1/2_	28.7 min
The area under the curve	AUC	2194 µM × min
Clearance	CL/F	1.37 mL min^-1^
Volume of distribution	V_d_/F	56.5 mL
Elimination rate	λ_z_	0.024 min^-1^

**Table 2:** Tissue/serum concentration ratio for PZ-DHA in Balb/c female mice.

Time (min)	Tissue/serum ratio of PZ-DHA (Mean ± SD)				
	Liver/serum	Lungs/serum	Kidneys/serum	Spleen/serum	Brain/serum
15	0.14 ± 0.06	3.33 ± 1.51	0.66 ± 0.03	3.29 ± 1.56	0.51 ± 0.17
30	0.16 ± 0.08	2.04 ± 1.09	1.29 ± 0.22	3.17 ± 0.72	0.64 ± 0.11
60	4.68 ± 2.02*	1.05 ± 0.014	0.81 ± 0.34	4.18 ± 1.01	0.95 ± 0.68
90	2.69 ± 2.17*	3.51 ± 2.44	0.89 ± 0.26	5.24 ± 1.75	1.01 ± 0.19
120	1.03 ± 0.73	3.82 ± 1.74	2.71 ± 1.41*	10.86 ± 8.42	1.31 ± 0.67
240	0.99 ± 0.23	4.97 ± 1.37	2.71 ± 1.08*	16.15 ± 5.98*	1.94 ± 0.75*

Tissue/serum concentration ratios for PZ-DHA were compared at each time point, considering 15 min as the baseline (n = 4). *p < 0.05. (Liver/serum *p* = 0.0004; Kidneys/serum *p* = 0.002; Spleen/serum *p* = 0.003; Brain/serum *p* = 0.01).

## Discussion

The rationale for conjugating PZ with DHA through an acylation reaction was to improve the cellular uptake and stability of PZ and DHA, respectively. This hypothesis was tested by conducting cellular uptake experiments of PZ, DHA, and PZ-DHA using mammary carcinoma cells and non-malignant mammary epithelial cells. The metabolism and pharmacokinetics of PZ-DHA were also investigated using in vitro and in vivo pharmacokinetic models.

PZ-DHA was taken-up by all tested mammary carcinoma cells (MDA-MB-231, MDA-MB-468, 4T1, and MCF-7) and non-malignant mammary epithelial cells (MCF-10A) when treated with a sub-cytotoxic concentration (20 µM). Quercetin, a commonly used internal standard in UPLC-ESI–MS/MS analysis of flavonoids, was used to determine the % recovery of test compounds, which was 53.27%. The metabolism of DHA is highly cell type-dependent. Park et al. showed that, following initial metabolism, DHA is esterified into cell lipids and retroconversion of DHA (22: 6n-3) to EPA (20: 5n-3) is five- to six-fold greater in non-neural cells compared to neural cells^22^. Furthermore, Parks et al. demonstrated long-term stable intake of DHA by erythrocytes^23^. Another study conducted using bovine aortic endothelial cell cultures shows that DHA is taken up by endothelial cells; however, the majority of DHA is incorporated into phospholipids^24^. Incubation with 20 µM ^13^C-DHA (22: 6n-3) shows cellular uptake by MCF-7 and HepG2 epithelial cells up to 24 h, as well as retroconverted product, ^13^C-eicosapentaenoic acid (20:5n-3)^22^. However, little is known about the stability and intracellular availability of DHA with prolonged incubation. On the other hand, DHA undergoes extensive auto-oxidation, oxidative degradation, and lipid peroxidation, resulting in a number of oxidative products such as mono/poly hydroxylated-DHA and epoxides^25–31^. Therefore, it is possible that DHA was also readily taken up by all mammary carcinoma/mammary epithelial cells used in the current study, but the detection was low as a result of its incorporation into phospholipids and other compartments of the cells and/or extensive degradation. Furthermore, it is important to note that a physiologically relevant concentration of DHA was used in the current study^32,33^. However, PZ-DHA showed a stable abundance throughout the experimental period (72 h), suggesting that conjugation of PZ with DHA increases the stability and availability of both PZ and DHA, which may lead to increased biological activity. Collectively, PZ-DHA increased the intra-cellular availability of PZ by at least 200–300-fold and DHA by 20–200-fold in mammary carcinoma cells. We have previously shown that PZ-DHA (20 µM) significantly inhibits the migration and invasion of different types of mammary carcinoma cells, including MDA-MB-231 triple-negative breast cancer cells and 4T1 murine mammary carcinoma cells^21^. The enhanced cellular accumulation of PZ and DHA when administered as PZ-DHA could explain why PZ-DHA was more effective than PZ or DHA alone. Furthermore, we have shown that PZ-DHA selectively kills mammary carcinoma cells (MDA-MB-231, MDA-MB-468, 4T1, and MCF-7) while having minimal harmful effects on non-malignant mammary epithelial cells (MCF-10A)^18^. Since the accumulation of PZ, DHA, and PZ-DHA are similar in malignant and non-malignant cells, the selective cytotoxic action is unlikely due to altered drug uptake and/or efflux. However, it is possible that PZ-DHA binds to the inner leaflet of the phospholipid bi-layer of the cell membrane and does not move into the cell interior of non-malignant cells because of differences in lipid and fatty acid composition of the phospholipid bi-layer of malignant and non-malignant cell membranes^34–36^. Interestingly, PZ-DHA uptake was not statistically different between all tested cell lines, suggesting that PZ-DHA uptake is not cell type-dependent.

In vitro phase I and phase II metabolism of PZ-DHA was studied using freshly prepared liver microsomal enzymes from the livers of C57BL/6 male mice. Three major NADPH-dependent phase I bio-transformations (hydrolysis, hydroxylation, and epoxidation) of PZ-DHA were identified. No further metabolism of DHA was recorded, suggesting a rapid degradation and/or accumulation of DHA into cellular compartments such as the phospholipid bilayer when hydrolyzed from PZ-DHA. Our previous work showed that PZ and DHA inhibits mammary cell carcinoma migration and invasion but to a lesser extent than PZ-DHA^21^. Therefore, it is possible that these metabolites may also contribute to the pharmacological effect of PZ-DHA in cell cultures and in mice. Phase II conjugation reactions typically serve as detoxifying reactions^9^ but can also produce metabolites that are more potent or toxic than the parent compounds. In some circumstances, phase II conjugated metabolites are less or equally potent as parent compounds, yet have improved pharmacological activity because of their slower systemic clearance and longer elimination half-lives. Manach et al. (1998) showed that 3′-*O*-methylquercetin, quercetin-3-*O*-sulfate, and quercetin glucuronides retain their antioxidant activities by prolonging the lag phase of the antioxidant activity^11^. Another study shows that glucuronide conjugates of quercetin (quercetin-4′-glucuronide, quercetin-3′-glucuronide, quercetin-7-glucuronide, and quercetin-3-glucuronide) act as potent xanthine oxidase and lipoxygenase inhibitors in vitro^37^. Although the composition of the microsomal enzyme mixture may vary from one mouse strain to another, this study identifies for the first time potential in vitro metabolites of PZ-DHA.

The pharmacokinetics of flavonoids is a complicated area of study. In plants, flavonoids exist as glucosides, except for catechins. It is, therefore, debatable whether flavonoids are better absorbed as aglycones or glucosides. In any case, the intestinal absorption of ingested flavonoids is low. It is possible that a fraction of PZ-DHA absorbed into the systemic circulation may also exist in a serum protein-bound form. In general, flavonoids show a high affinity for plasma proteins, as well as high distribution into fat tissue^38–40^. Boulton et al. showed that 99.1% of quercetin is bound to human plasma proteins, leaving only 0.9% of quercetin freely available in the plasma. Another study showed that the binding of four flavonoids (orientin, vitexin, cynaroside, and quercetin) to plasma proteins ranged from 74 to 89%^41^. Flavonoids accumulate in the liver, lungs, and kidneys following parenteral or oral administration^42,43^. Substances that are administered by intraperitoneal injection are mainly absorbed via the superior and inferior mesenteric veins, which drain into the portal vein^44,45^. Therefore, following intraperitoneal administration, PZ-DHA must pass through the liver before entering the systemic circulation and, as a result, may undergo hepatic metabolism to generate phase II metabolites, which would also affect the amount of intact PZ-DHA in blood. This was evident by the presence of the PZ-DHA metabolites in the liver, kidneys, and lungs within 30 min of intraperitoneal administration of PZ-DHA. In our recently published study, we have shown that intraperitoneal administration of PZ-DHA (100 mg/kg every 2 days for 10 days) inhibits tumor growth and metastasis of 4T1 breast cancer cells in female Balb/c mice^21^. The pharmacokinetic analysis demonstrates a peak concentration of PZ-DHA in the serum of 23.4 μM, which is very close to the concentration (20 μM) that significantly inhibits breast cancer cell migration and invasion in vitro. Thus, these results confirm that pharmacologically active concentrations of PZ-DHA are attained in vivo when PZ-DHA is administered at the 100 mg/kg dose. Despite the rapid half-life of elimination (28 min) of PZ-DHA, the extensive tissue distribution of PZ-DHA (large V_d_ 57 mL) and increased accumulation in tissues (other than the liver) over time could contribute to the previously observed reduction in breast tumour volume and lung metastasis with the 100 mg/kg PZ-DHA dose. PZ-DHA and its tri-methylated-metabolite accumulated in the kidneys during the serum elimination phase of PZ-DHA, signifying potential renal excretion of PZ-DHA both in intact and methylated forms. Access of drugs/xenobiotics to the brain is strictly controlled by the blood–brain barrier. Multiple studies have shown that methylated and glucuronide conjugates of flavonoids cross the blood–brain barrier and enter the brain^46–48^. The availability of PZ-DHA in the brain suggests that flavonoid fatty acid esters cross the blood–brain barrier without needing to undergo phase II metabolism. In addition, some studies have discussed the clinical application of flavonoid conjugates^49,50^. While these experiments show PZ-DHA accumulation in the liver and kidney, our previous toxicological studies confirmed that PZ-DHA does not show liver or kidney toxicity in Balb/c female mice when given at 100 mg/kg body weight via intraperitoneal injections^21^.

**Figure 6:**
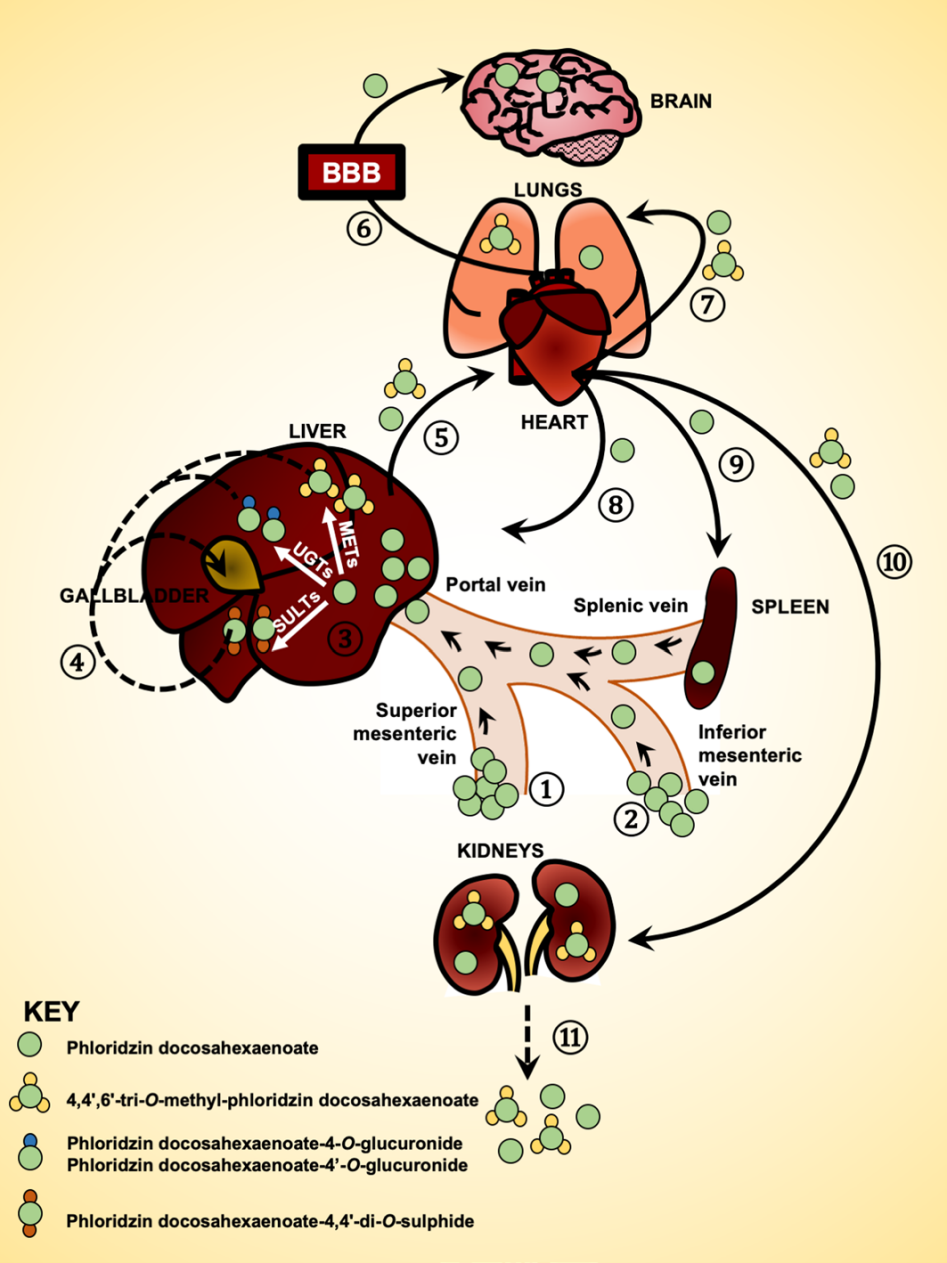
Proposed scheme for the fate of PZ-DHA upon intraperitoneal administration into Balb/c female mice. Absorption of intraperitoneally administered PZ-DHA via ① superior mesenteric vein and ② inferior mesenteric vein; ③ Phase II metabolism of PZ-DHA in the liver; ④ Biliary secretion and entero-hepatic circulation of PZ-DHA and its metabolites; Distribution of PZ-DHA and its metabolites via ⑤ inferior vena cava, ⑥ left and right carotid arteries, ⑦ bronchial arteries branched from the descending aorta; ⑧ hepatic artery branched from descending aorta, ⑨ splenic artery, and ⑩ renal arteries branched from descending aorta; ⑪ PZ-DHA may be excreted via kidneys. Solid-lined arrows (→) indicate confirmed processes and dashed-line arrows (⇢) indicate suggested potential processes, which need to be confirmed in future studies. *BBB* blood brain barrier; *METs* methyltransferases; *UGTs* UDP-glucuronosyltransferase; *SULTs* sulphotransferases.

The proposed scheme for absorption, distribution, metabolism, and excretion of PZ-DHA following intraperitoneal administration is outlined in Fig. 6. Taken together, findings from in vivo pharmacokinetic experiments and related UPLC-ESI–MS/MS analysis shows that, following intraperitoneal administration, PZ-DHA is readily absorbed into the systemic circulation and distributed widely throughout the body. PZ-DHA undergoes both phase I and phase II metabolism in the liver, and is then eliminated, possibly by conjugation followed by renal excretion. The presence of PZ-DHA and its metabolites in multiple organs suggest the potential pharmacological use of PZ-DHA in many ailments. Inhibition of lung metastases following intraperitoneal administration of PZ-DHA could partly be attributed to its lung distribution. The relatively high accumulation of PZ-DHA in the brain suggests that future studies should assess the potential use of PZ-DHA for central nervous system tumors, given that passage of effective drugs across the blood–brain barrier is a significant limitation to current treatment. Although the ideal results of a cell uptake assay would have been obtained by using a radioactive isotype-labeled PZ-DHA, the LC–MS-MS method has also been successfully used to quantify fatty acids in biological samples^51,52^. We have used two different mouse strains in the current study. Although there can be mouse strain-dependent differences in drug metabolism, studies have shown a good general agreement of pharmacokinetics between different mouse strains^53–55^. Furthermore, given the accumulation of PZ-DHA in the spleen, its effects on tumor-related immune responses should be explored. Lastly, renal drug excretion experiments need to be performed to confirm and extend PZ-DHA kidney accumulation data.

## Materials and methods

### Chemicals and reagents

Bradford assay reagent was purchased from Bio-Rad Laboratories, Inc. (Mississauga, ON, Canada). Alamethicin and UDP-glucuronic acid (UDPGA) were obtained from Corning Life Sciences (Tewkbury, MA, USA). Dulbecco’s Modified Eagle’s Medium (DMEM), F12/DMEM, fetal bovine serum (FBS), L-glutamine, N-2-hydroxyethylpiperazine-N′-2-ethanesulfonic acid (HEPES), penicillin–streptomycin (Pen-strep) solution, TryPLE Express, and trypsin [0.25% with EDTA (1 ×)] were obtained from Life Technologies Inc. (Burlington, ON, Canada). Docosahexaenoic acid (DHA) was purchased from Nu-Chek Prep Inc (Elysian, MN, USA). Human epidermal growth factor (EGF) was purchased from PeproTech (Rocky Hill, NJ, USA). NADPH was obtained from Roche Diagnostics (Laval, QC, Canada). Bovine insulin, bovine serum albumin (BSA), hydrocortisone, 3′-phosphoadenosine-5′-phosphosulfate (PAPS), phloridzin (PZ), quercetin, S-adenosylmethionine (SAM) were purchased from Sigma-Aldrich (Oakville, ON, Canada).

### Cell cultures and cell culture conditions

MDA-MB-231 cells were provided by Dr. S. Drover (Memorial University of Newfoundland, St. John’s, NL, Canada). MCF-7 cells were provided by Dr. R. Robey (National Cancer Institute, Bethesda, MD, USA). 4T1 mouse mammary carcinoma cells were obtained from Dr. D. Waisman (Dalhousie University, Halifax, NS, Canada). MDA-MB-468 cells were obtained from Dr. P. Lee (Dalhousie University, Halifax, NS, Canada). Mammary carcinoma cell lines were authenticated by short tandem repeat analysis conducted by American Type Culture Collection (ATCC; Manassas, VA, USA) (MDA-MB- 231, MDA-MB-468, and MCF-7) and DDC Medical DNA Diagnostic Center (Fairfield, OH, USA) (4T1). DMEM basal medium supplemented with 10% v/v heat-inactivated FBS, 5 mM HEPES (pH 7.4), 2 mM l-glutamine, 100 U/mL penicillin and 100 μg/mL streptomycin was used as the growing medium (cDMEM) for all mammary carcinoma cell lines^18^. MCF-10A cells were obtained from Dr. P. Marcato (Dalhousie University, Halifax, NS, Canada) and grown in F12/DMEM (1:1) medium supplemented with 10% horse serum, 0.02 μg/mL EGF, 0.5 μg/mL hydrocortisone, 10 μg/mL bovine insulin, 100 U/mL penicillin and 100 μg/mL streptomycin^18^. All malignant cell cultures were maintained at 37 °C in a humidified incubator supplied with 10% CO_2_ and MCF-10A cells were maintained at 37 °C in a humidified incubator supplied with 5% CO_2_^18^.

### Mice

All experiments involving mice were approved by the Dalhousie University Committee on Laboratory Animals and were conducted in accordance with the guidelines set out by the Canadian Council for Animal Care. Six to eight-weeks-old C57BL/6 and Balb/c mice were purchased from Charles River Canada (Lasalle, QC, Canada) and housed in the Carlton Animal Care Facility, Tupper Medical Building of Dalhousie University. Mice were fed with regular rodent chow, and water was supplied ad libitum^21^.

### Preparation of mouse hepatic microsomal enzyme extractions

Mouse hepatic microsomes were freshly isolated from the livers of male C57BL/6 mice, and the entire extraction procedure was carried out at 4 °C. Mouse livers were minced and homogenized in KCl/sucrose phosphate buffer (0.154 M KCl, 0.25 M sucrose in 50 mM potassium phosphate buffer (K_2_HPO_4_/KH_2_PO_4_) (PPB), pH 7.5). Cell/tissue debris was separated out by centrifuging homogenized livers at 12,000×*g* for 22 min at 4 °C. The supernatant was collected and centrifuged at 100,000×*g* for 70 min at 4 °C. The pellet was gently rinsed using 50 mM PPB and resuspended in glycerol/phosphate buffer (20% glycerol (v/v), 80% 0.1 M PPB pH 7.5). The protein concentration was determined using a Bradford assay (R^2^ = 1.00), and microsomes were stored at − 80 °C.

### Cellular uptake assays for PZ, DHA, and PZ-DHA

Cells were seeded at a density of 3 × 10^5^ cells (MDA-MB-231, MDA-MB-468, MCF-7, and MCF-10A) or 1 × 10^5^ cells (4T1) /T-75 flask. The cells were cultured overnight to promote cell adhesion. Adherent cells were treated with a sub-cytotoxic concentration (20 μM) of PZ, DHA or PZ-DHA and cultured for 72 h at 37 °C. The supernatant was carefully removed, and cell monolayers were washed with cold PBS. Cells were harvested using TrypLE Express and washed thoroughly with cold PBS. The cell pellet was resuspended in 3 mL of cold acetone containing 0.05 mg/mL quercetin as the internal standard, and incubated overnight at 4 °C. Cell lysates were collected by centrifugation at 14,000×*g* for 15 min at 4 °C and samples were concentrated by evaporating acetone under a stream of nitrogen gas. Dry pellets were reconstituted in methanol and filtered. The sample was analyzed using a UPLC/ESI/MS system. The percentage cell uptake of the total dose was quantified using standard curves of PZ (R^2^ = 0.99), DHA (R^2^ = 0.98) or PZ-DHA (R^2^ = 0.99) made in methanol.

### Determination of in vitro metabolites of PZ-DHA

Potential i*n vitro* phase I and phase II metabolites of PZ-DHA were detected by incubating PZ-DHA in the presence of freshly-prepared mouse hepatic microsome preparations. Phase I metabolites were determined by incubating 1.1 µM PZ-DHA with 100 mM NADPH in the presence of mouse hepatic microsomes for 1 h at 37 °C. Phase II methylation, glucuronidation, and sulphation of PZ-DHA was determined by incubating 1.1 µM PZ-DHA with 1 mM SAM, 2 mM UDPGA, and 0.5 mM PAPS, respectively, in the presence of freshly prepared mouse hepatic microsome for 1 h at 37 °C (Supplementary information Tables S1–S4). To assess the efficiency of the metabolic process, controls (no PZ-DHA control, no mouse hepatic microsome control and no co-factor control) were incubated in parallel to the test. Reactions were terminated by adding a mixture of 4 mL ice-cold acetonitrile:acetone (80:20) containing 0.05 mg/mL quercetin as the internal standard. Proteins were precipitated by incubating the reaction mixture at 4 °C overnight and separated by centrifuging at 14,000 × g for 15 min at 4 °C. Supernatants were concentrated under nitrogen flush, filtered and PZ-DHA and/or PZ-DHA metabolites were identified using a UPLC-ESI–MS/MS system (Waters Limited, Mississauga, ON, Canada).

### Liquid chromatography/mass spectrometry

PZ-DHA and/or PZ-DHA metabolites in malignant/non-malignant epithelial cell lysates, in vitro microsome enzyme reaction mixtures, and mouse serum aliquots were determined using UPLC-ESI–MS/MS instrument (Waters Limited, Mississauga, ON, Canada). All mass spectroscopic analyses were performed using mass scan (full-scan, identification of unknowns) and single ion monitoring (SIM; quantification of PZ, DHA, PZ-DHA, and quercetin) modes of a Waters H-class UPLC separations module coupled with a Micromass Quattro micro API MS/MS system and MassLynx V4.0 control software. An Allure biphenyl column (100 mm × 2.1 mm, 1.8 μm, Restek Pure Chromatography, Bellefonte, PA, USA) and a flow rate of 0.3 mL/min were employed. ESI- mode with a capillary voltage of 3000 V and a nebulizer gas (N_2_) temperature of 375 °C were used. A volume of 2 µL sample was injected.

### Mass spectrometry analysis of PZ-DHA and metabolites from in vivo studies

Pharmacokinetic parameters and in vivo generation of PZ-DHA metabolites were studied using Balb/c female mice. Mice were randomly divided into six groups (n = 4/group) and PZ-DHA (100 mg/kg) was administered by intraperitoneal injection. For each mouse, a single blood collection was performed by cardiac puncture at one of six different time points (t = 15, 30, 60, 90, 120, and 240 min). At the end of blood collection, mice were euthanized by cervical dislocation and organs (liver, lungs, kidneys, spleen, and brain) were harvested. Blood samples were allowed to clot by leaving undisturbed at room temperature for 1 h, and serum was separated by centrifuging at 2000×*g* for 10 min at 4 °C. The serum was carefully removed and stored on ice until further processing. Serum proteins were precipitated by incubating an aliquot of serum with a mixture of acetonitrile:acetone (80:20) containing 0.1 mg/mL quercetin (internal standard) at 4 °C overnight. The supernatant was separated by centrifuging at 14,000×*g* for 10 min at 4 °C. Excess solvent was evaporated using nitrogen flush and mixed with methanol and filtered. PZ-DHA-metabolites in the filtrate were identified using a UPLC-ESI–MS/MS (Waters Limited, Mississauga, ON, Canada). Organs were homogenized in PBS (1:5 w/v). Supernatants were collected by centrifugation at 14,000 × g for 15 min at 4 °C. Proteins were precipitated by incubating in acetonitrile:acetone (80:20) containing 0.1 mg/mL quercetin (internal standard) at 4 °C overnight. Supernatants were treated as explained above, and PZ-DHA and/or PZ-DHA-metabolites were identified.

### Assessment of PZ-DHA pharmacokinetics

A calibration curve (R^2^ = 0.99) generated using a standard series of PZ-DHA concentrations prepared in mouse serum (spiked with 0.1 mg/mL quercetin) was used for the quantification of PZ-DHA. The average PZ-DHA concentrations were plotted against time. The maximum serum concentration of serum (C_max_) and the time to reach C_max_ (T_max_) were read directly from the graph. The linear trapezoidal method was used to calculate the area under the curve (AUC) from t = 0 to infinity, the biological half-life of PZ-DHA (t_1/2_), determined using the plotted curve in Graphpad Prism software (version 5; La Jolla, CA, USA). The clearance following IP administration was determined by non-compartmental analysis using the relationship CL/F = D/AUC. The elimination rate constant λ was determined by linear regression of the elimination phase of the semi-log transformed concentration versus time curve. The t_1/2_ was calculated using the formula 0.693/λ and the volume of distribution V_d_/F from the formula V_d_/F = CL/(F*λ).

### Data and statistical analysis

All statistical and graphical analyses were performed using GraphPad Prism software (version 5). One-way ANOVA followed by Tukey’s multiple means comparison method was used to compare the difference among PZ, DHA, and PZ-DHA. *P* < 0.05 was considered to be a statically significant change.

## Supplementary Information

Supplementary Information
