# Supplementary material for: Metabolism and pharmacokinetics of a novel polyphenol fatty acid ester phloridzin docosahexaenoate in Balb/c female mice
Source: Sci Rep. 2020 Dec 7;10:21391. doi: 10.1038/s41598-020-78369-0 (PMC7721897; doi:10.1038/s41598-020-78369-0)
Supplement: Supplementary file 1 — Supplementary Information [file 41598_2020_78369_MOESM1_ESM.pdf]

SUPPLEMENTARY INFORMATION (TABLES)

Metabolism and pharmacokinetics of a novel polyphenol fatty acid ester phloridzin docosahexaenoate in Balb/c female mice

Wasundara Fernando[1], Kerry B. Goralski[2,3,6,8], David W. Hoskin[1,4,5], H. P. Vasantha Rupasinghe[1,7]*

Table S1. Reaction mixture used for the determination of *in vitro* phase I metabolism of PZ-DHA.

DMSO: Dimethyl sulfoxide; NADPH: Nicotinamide adenine dinucleotide phosphate; PPB: Potassium phosphate buffer; PZ-DHA: Phloridzin docosahexaenoate.

Sample ID	Components of the reaction mixture (*Final concentration)			
	PZ-DHA in DMSO (*300 µM) (µL)	Mouse hepatic microsomes (*3 mg/mL) (µL)	NADPH in PPB (*100 mM) (µL)	PPB (µL)
Test	6	334	100	1560
Control 1	-	334	100	1566
Control 2	6	-	100	1894
Control 3	6	334	-	1660
Control 4	6	-	-	1994

Table S2. Reaction mixture used for the determination of *in vitro* phase II methylation of PZ-DHA.

DMSO: Dimethyl sulfoxide; PZ-DHA: Phloridzin docosahexaenoate; SAM: S-(5′-Adenosyl)-L-methionine chloride.

Sample ID	Components of the reaction mixture (*Final concentration)			
	PZ-DHA in DMSO (*300 µM) (µL)	Mouse hepatic microsomes (*3 mg/mL) (µL)	SAM in purified water (*1 mM) (µL)	Purified water (µL)
Test	6	334	200	1460
Control 1	-	334	200	1466
Control 2	6	-	200	1894
Control 3	6	334	-	1660

Table S3. Reaction mixture used for the determination of *in vitro* phase II glucuronidation of PZ-DHA.

DMSO: Dimethyl sulfoxide; PZ-DHA: Phloridzin docosahexaenoate; UDPGA: UDP-glucuronic acid.

Sample ID	Components of the reaction mixture (*Final concentration)				
	PZ-DHA in DMSO (*300 µM) (µL)	Mouse hepatic microsomes (*6 mg/mL) (µL)	UDPGA – Sol A (*2 mM) (µL)	Buffer with Alamethicin – Sol B (*25 µg/mL) (µL)	Purified water (µL)
Test	6	334	160	400	1100
Control 1	-	334	160	400	894
Control 2	6	-	160	400	1434
Control 3	6	334	-	400	1260

Table S4. Reaction mixture used for the determination of *in vitro* phase II sulphation of PZ-DHA.

DMSO: Dimethyl sulfoxide; PAPS: Adenosine 3′-phosphate 5′-phosphosulfate lithium salt hydrate; PZ-DHA: Phloridzin docosahexaenoate.

Sample ID	Components of the reaction mixture (*Final concentration)			
	PZ-DHA in DMSO (*300 µM) (µL)	Mouse hepatic microsomes (*3 mg/mL) (µL)	PAPS in purified water (*0.5 mM) (µL)	Purified water (µL)
Test	6	334	200	1460
Control 1	-	334	200	1466
Control 2	6	-	200	1894
Control 3	6	334	-	1660
